# Tracking of total sedentary time and sedentary patterns in youth: a pooled analysis using the International Children’s Accelerometry Database (ICAD)

**DOI:** 10.1186/s12966-020-00960-5

**Published:** 2020-05-18

**Authors:** Evi van Ekris, Katrien Wijndaele, Teatske M. Altenburg, Andrew J. Atkin, Jos Twisk, Lars B. Andersen, Kathleen F. Janz, Karsten Froberg, Kate Northstone, Angie S. Page, Luis B. Sardinha, Esther M. F. van Sluijs, Mai Chinapaw, On behalf of the International Children’s Accelerometry Database (ICAD) Collaborators, L. B. Andersen, S. Anderssen, A. J. Atkin, G. Cardon, R. Davey, U. Ekelund, D. W. Esliger, P. Hallal, B. H. Hansen, K. F. Janz, S. Kriemler, N. Møller, K. Northstone, A. Page, R. Pate, J. J. Puder, J. Reilly, J. Salmon, L. B. Sardinha, L. B. Sherar, E. M. F. van Sluijs

**Affiliations:** 1 Department of Public and Occupational Health, Amsterdam Public Health research institute, VU University Medical Center, van der Boechorststraat 7, 1081BT Amsterdam, the Netherlands; 2 MRC Epidemiology Unit, University of Cambridge, Cambridge, UK; 3 Centre for Diet and Activity Research (CEDAR), MRC Epidemiology Unit, University of Cambridge, Cambridge, UK; 4 School of Health Sciences, Faculty of Medicine and Health Sciences, University of East Anglia, Norwich, UK; 5 Department of Epidemiology and Biostatistics, VU University Medical Center, Amsterdam, the Netherlands; 6 Faculty of Teacher Education and Sport, Western Norway University of Applied Sciences, Sogndal, Norway; 7 Department of Health and Human Physiology, University of Iowa, Iowa City, USA; 8 Research of Childhood Health, University of Southern Denmark, Odense, Denmark; 9 Department of Population Health Sciences, Bristol Medical School, University of Bristol, Bristol, UK; 10 Centre for Exercise, Nutrition and Health Sciences, University of Bristol, Bristol, UK; 11 NIHR Bristol Biomedical Research Centre, University Hospitals Bristol NHS FoundationTrust and University of Bristol, Bristol, UK; 12 Exercise and Health Laboratory, CIPER, Faculdade de Motricidade Humana, Universidade de Lisboa, Lisbon, Portugal

**Keywords:** ICAD, Sedentary time, Accelerometer, Tracking, Children, Adolescents, Objective assessment

## Abstract

**Background:**

To gain more understanding of the potential health effects of sedentary time, knowledge is required about the accumulation and longitudinal development of young people’s sedentary time. This study examined tracking of young peoples’ total and prolonged sedentary time as well as their day-to-day variation using the International Children’s Accelerometry Database.

**Methods:**

Longitudinal accelerometer data of 5991 children (aged 4-17y) was used from eight studies in five countries. Children were included if they provided valid (≥8 h/day) accelerometer data on ≥4 days, including ≥1 weekend day, at both baseline and follow-up (average follow-up: 2.7y; range 0.7–8.2). Tracking of total and prolonged (i.e. ≥10-min bouts) sedentary time was examined using multilevel modelling to adjust for clustering of observations, with baseline levels of sedentary time as predictor and follow-up levels as outcome. Standardized regression coefficients were interpreted as tracking coefficients (low: < 0.3; moderate: 0.3–0.6; high: > 0.6).

**Results:**

Average total sedentary time at study level ranged from 246 to 387 min/day at baseline and increased annually by 21.4 min/day (95% confidence interval [19.6–23.0]) on average. This increase consisted almost entirely of prolonged sedentary time (20.9 min/day [19.2–22.7]). Total (standardized regression coefficient (B) = 0.48 [0.45–0.50]) and prolonged sedentary time (B = 0.43 [0.41–0.45]) tracked moderately. Tracking of day-to-day variation in total (B = 0.04 [0.02–0.07]) and prolonged (B = 0.07 [0.04–0.09]) sedentary time was low.

**Conclusion:**

Young people with high levels of sedentary time are likely to remain among the people with highest sedentary time as they grow older. Day-to-day variation in total and prolonged sedentary time, however, was rather variable over time.

## Background

Children and adolescents spend the main part of their waking hours sedentary [1–3], and their sedentary time is consistently found to increase with age [4, 5]. Cooper et al. [6] for example reported in their cross-sectional study that daily sedentary time of adolescents aged 17–18 years is approximately 25% higher compared to children aged 5–6 years. In young people, some evidence indicates that sedentary time, and in particular TV viewing time, is associated with an increased risk for overweight and decreased fitness [7] but evidence for health effects of young people’s sedentary time is in general inconclusive [7, 8]. In order to gain more understanding of potential health effects of sedentary time, more knowledge is needed about the accumulation of sedentary time and how levels of sedentary time evolve over time. Childhood may be a critical period for the development of sedentary behaviour habits [4, 9]. This highlights the need for a better understanding of how young people’s sedentary time tracks longitudinally. Tracking of sedentary time refers to the degree in which current time spent sedentary can predict time spent sedentary at a later timepoint [10]. If tracking is high, people are likely to retain their rank for sedentary time within the population over time.

Systematic reviews typically find moderate tracking of young people’s sedentary behaviour [9, 11, 12]. However, the existing literature presents two main limitations. First, most studies examine tracking of TV viewing and screen time [9, 11, 12], which are not representative of total sedentary time [13]. Only four previous studies examined tracking of objectively-assessed total sedentary time in young children, children or adolescents [4, 14–16] and found fair-to-moderate tracking of sedentary time, except for the study of Kelly et al. [16] in which low tracking of sedentary time was found in young children during the preschool-to-school transition period. Large scale international studies examining tracking of objectively measured sedentary time, using a diverse study population are currently lacking. A second limitation of the exisiting literature is the limited evidence on tracking of the pattern in which total sedentary time is accumulated. Recent experimental [17, 18] and epidemiological studies [19–21] in young people suggest that not only total sedentary time but also the extent to which sedentary time is accumulated in prolonged bouts (i.e. prolonged sedentary time) might be important for health. We have identified only two previous studies examining tracking of sedentary bouts; both of which examined the number of sedentary bouts [4, 15]. Janz et al. [15] found that tracking of the number of sedentary bouts (defined as bouts ≥5 min) was slighty lower than tracking of total sedentary time, although tracked moderately. Janssen et al. [4] found that tracking coefficients for the number of bouts ranged between 0.06–0.52, depending on bout length, age of children and follow-up duration. Tracking of the total time accumulated in bouts has not been examined before, but might be more directly related to health than the number of sedentary bouts per se [22]. Another approach to examine accumulation of sedentary time is to look at variation in sedentary time between multiple days (i.e. day-to-day variation). Generally, the average sedentary time is calculated over multiple days, but no previous studies examined tracking of day-to-day variation in sedentary time. Insight in how sedentary time is accumulated throughout the week and how this day-to-day variation tracks over time may be important to inform interventions targeting sedentary behaviour: e.g. by providing more precise intervention settings and time windows.

This present study therefore aims to examine tracking of total sedentary time, prolonged sedentary time (defined as time accumulated in sedentary bouts ≥10 min) and day-to-day variation in total and prolonged sedentary time during childhood, in a large international sample. Previous research indicates that sedentary time and the rate of change in sedentary time may be different for boys and girls and during different phases of childhood and adolescence (e.g. during childhood and the transition from childhood to adolescence) [4, 6]. Therefore, the moderating effects of gender and age group were examined.

## Methods

### Study design and participants

Data were obtained from the International Children’s Accelerometry Database (ICAD, http://www.mrc-epid.cam.ac.uk/research/studies/icad). The ICAD was established to pool data from studies conducted around the world using the Actigraph accelerometer to objectively measure young people’s sedentary time and physical activity. A detailed description of the ICAD including aims, design and methods can be found elsewhere [23]. The full database contains data of approximately 37,000 children (aged 3–18 years) from 20 studies in 10 countries. For the purpose of the present study, data were extracted from eight observational studies with longitudinal data available (total *N* = 14,098; Table 1 [24–32]). The eight included studies were conducted in five different countries and data were collected between 1997 and 2011. Five studies had two waves of data collection, two studies had three waves and one study had four waves of data available. All included studies obtained relevant ethical approval and appropriate consent.

**Table 1:** Study and participant characteristics

	Wave	ALSPAC	EYHS Denmark	EYHS Portugal	HEAPS	IBDS	PEACH	SPEEDY	CLAN^a^
Country		UK	Denmark	Portugal	Australia	US	UK	UK	Australia
Number of waves		2 waves	2 waves	2 waves	2 waves	4 waves	2 waves	3 waves	3 waves
Average follow-up duration (years)	W1-W2	2.1 ± 0.3	6.0 ± 0.3	7.2 ± 0.2	3.1 ± 0.2	2.9 ± 0.5	1.1 ± 0.1	1.0 ± 0.04	3.0 ± 0.06
	W1-W3					5.4 ± 0.5		4.1 ± 0.1	4.9 ± 0.2
	W1-W4					7.4 ± 0.5			
Data-collection period	W1	All months 2003–2005	All months 1997–1998	January – June 2000	February – December 2002–2003	September –November 1998–2000	January – November 2006–2008	February – July 2007	March–December 2001
	W2	All months 2005–2007	All months 2003–2004	January – July 2007–2008	May – December	September –November 2000–2004	All, 2007–2009	March – July 2008	July – November 2004
	W3					September –November 2003–2005		April–September 2011	May–November 2006
	W4					September–December 2005–2007			
Number of participants with baseline accelerometer data / valid baseline accelerometer data	W1	6085 / 5455	529 / 283	1218 / 631	1389 / 1040	437 / 329	1269 / 816	2009 / 1708	1162 / 1022
Number of participants included in analysis	W1	2992	122	33	226	306	386	663	428
	W2	2992	122	33	226	227	386	565	361
	W3					267		275	324
	W4					213			
Gender (% boys)	W1	46.6	41.0	36.4	51.8	46.4	39.9	43.4	46.3
Mean age and range (years)	W1	11.7 ± 0.2 (10.6–13.6)	9.6 ± 0.4 (8.6–10.5)	9.7 ± 0.3 (9.1–10.4)	8.1 ± 2.6 (5.0–12.6)	5.6 ± 0.5 (4.7–7.5)	10.9 ± 0.4 (10.1–11.8)	10.2 ± 0.3 (9.5–10.8)	9.5 ± 2.7 (5.5–15.0)
	W2	13.8 ± 0.2 (12.6–14.8)	15.7 ± 0.4 (14.9–16.8)	16.9 ± 0.31 (16.3–17.5)	11.2 ± 2.6 (8.1–15.9)	8.6 ± 0.5 (7.6–10.7)	12.0 ± 0.4 (11.1–12.8)	10.8 ± 0.4 (10.0–12.0)^b^	12.5 ± 2.7 (8.5–15.9)
	W3					11.1 ± 0.3 (10.6–12.7)		14.3 ± 0.3 (13.7–14.9)	14.1 ± 2.6 (10.5–17.7)
	W4					13.0 ± 0.3 (12.5–14.4)			
BMI z-score	W1	0.31 ± 1.16	0.19 ± 0.98	0.44 ± 1.33	0.69 ± 0.98	0.37 ± 1.06	0.29 ± 1.17	0.37 ± 1.16	0.60 ± 1.04
	W2	0.20 ± 1.09	0.08 ± 0.86	0.22 ± 0.99	0.62 ± 0.99	0.65 ± 1.31	0.30 ± 1.17	NA.	0.60 ± 1.07
	W3					0.73 ± 1.31		0.25 ± 1.20	0.54 ± 1.04
	W4					0.65 ± 1.28			
Total sedentary time (minutes/day)	W1	370 ± 68	333 ± 101	366 ± 116	282 ± 73	246 ± 49	387 ± 64	371 ± 64	316 ± 79
	W2	425 ± 76	492 ± 72	544 ± 78	388 ± 96	318 ± 57	414 ± 69	391 ± 65	381 ± 92
	W3					356 ± 65		475 ± 73	446 ± 104
	W4					413 ± 73			
Prolonged sedentary time (minutes/day)	W1	158 ± 62	143 ± 103	183 ± 134	102 ± 45	81 ± 36	177 ± 61	172 ± 61	122 ± 57
	W2	209 ± 78	272 ± 90	366 ± 97	176 ± 90	118 ± 49	196 ± 63	191 ± 64	172 ± 79
	W3					148 ± 56		269 ± 81	237 ± 110
	W4					203 ± 73			
Day-to-day variation in total sedentary time (minutes/day)	W1	54 ± 19	53 ± 21	50 ± 24	48 ± 19	41 ± 19	55 ± 21	54 ± 21	51 ± 22
	W2	60 ± 22	68 ± 27	74 ± 37	56 ± 21	47 ± 21	60 ± 22	55 ± 19	57 ± 22
	W3					51 ± 22		64 ± 25	63 ± 27
	W4					57 ± 25			
Day-to-day variation in prolonged sedentary time (minutes/day)	W1	47 ± 19	45 ± 24	44 ± 23	40 ± 20	32 ± 17	48 ± 19	48 ± 20	43 ± 22
	W2	55 ± 23	63 ± 28	84 ± 34	51 ± 23	38 ± 20	53 ± 21	53 ± 20	52 ± 24
	W3					43 ± 20		63 ± 23	61 ± 30
	W4					57 ± 23			
MVPA^c^ (minutes/day) (median with (interquartile range))	W1	30 (24)	23 (25)	19 (33)	38 (28)	25 (18)	21 (17)	25 (21)	35 (23)
	W2	30 (26)	17 (20)	23 (22)	26 (23)	31 (24)	23 (20)	23 (21)	39 (30)
	W3					26 (26)		20 (21)	29 (24)
	W4					22 (24)			
Accelerometer wear time (minutes/day)	W1	792 ± 52	776 ± 56	783 ± 72	755 ± 64	768 ± 43	769 ± 60	773 ± 57	782 ± 65
	W2	792 ± 62	831 ± 56	820 ± 78	778 ± 66	786 ± 44	786 ± 70	773 ± 61	796 ± 65
	W3					783 ± 43		796 ± 63	803 ± 66
	W4					790 ± 57			

Data are reported as means ± standard deviations, unless otherwise stated. ^a^ Baseline wave was called CLASS; ^b^ calculated from self-reported age in years; ^c^ data for MVPA was not normally distributed and is reported as median with (interquartile range)

### Measurements

All studies used waist-worn uniaxial Actigraph accelerometers (models 7164, 71,256 and GT1M; Pensacola, FL, USA), which have acceptable validity for estimating sedentary time in youth [33, 34]. Raw accelerometer data was re-processed centrally (pampro, Github repository). Higher resolution data was reintegrated to 60 s epoch resolution, epochs with an intensity > 30,000 cpm were classified as non-valid and time between midnight and 7 am was discarded. Non-wear time was defined as bouts ≥60 min of consecutive zeros, allowing no tolerance time [35]. A day was defined as valid when the accelerometer was worn for ≥480 min (between 7 am and midnight). Participants were included in the analysis when they provided valid data for at least 4 days, including at least 1 weekend day, both at baseline and at least one follow-up assessment. Total sedentary time was calculated using a cut-point of < 100 counts per minute (CPM) [36, 37]. Prolonged uninterrupted sedentary time was defined as sedentary time accumulated in bouts of at least 10 consecutive minutes at < 100 CPM [35], allowing no tolerance for interruptions [38]. There is currently a lack of consensus on definitions of prolonged sedentary time. We based our decision to use ≥10 min as criteria of prolonged sitting on the paper of Altenburg et al. [35]. This paper pointed out that bouts of ≥20 min are very rare in children. Although bouts of ≥5 min are very common in children, it is unlikely that such short sedentary bouts have health effects. Therefore, we chose ≥10 min to define prolonged sedentary time.

Day-to-day variability in total sedentary time, and in prolonged uninterrupted sedentary time (min/day), was calculated as the sum of absolute differences between the values for each measurement day (*x*_i_) and the mean value ($\overline{x}$), divided by the number of days on which the accelerometer was worn (*N*) and was calculated for each individual participant.

$$ \frac{\sum \left|{x}_i-\overline{x}\right|}{N} $$

Moderate-to-vigorous intensity physical activity (MVPA) was calculated for descriptive purposes, using a cut-point of ≥3000 CPM [39]. Body mass index (BMI, in kg/m^2^) was calculated for descriptive reasons as well and expressed as z-scores (BMI z-score) based on gender and age using World Health Organisation reference data [40]. Information on participants’ body height and body weight was lacking from SPEEDY wave 2, but was available for all other studies and waves. BMI z-score was therefore not reported for SPEEDY wave 2.

### Statistical analyses

Study and wave specific descriptive characteristics were calculated using SPSS 20.0 (SPSS, Inc., Chicago, IL, USA). For all analyses, Individual patient data (IPD) meta-analyses were performed. Because the ICAD data is hierarchical, multilevel lineair regression modelling with a 4-level structure (country, study, child and repeated observations within the child) was used for all analyses to adjust the regression coefficients for clustering of observations. In order to estimate the annual change (expressed in minutes/day) in total sedentary time, prolonged sedentary time and their day-to-day variation, time was added to the multilevel model. The analyses were adjusted for wear time due to differences across children. For tracking analyses, baseline levels for sedentary time or its day-to-day variation were used as independent variables and the follow-up measurements were used as outcome. If there was more than one follow-up measurement available, data of all follow-up measurements were included in the model. Regression coefficients for baseline levels can be interpreted as longitudinal tracking coefficients. Tracking coefficients were standardized to maximize comparison with other tracking coefficients; standardized coefficients therefore range between 0 (no tracking) and 1 (perfect tracking). Tracking was defined as low when the standardized coefficient was < 0.3; moderate 0.3–0.6; and high > 0.6 [10, 41]. ‘No evidence of tracking’ was assigned when the association between baseline and follow-up levels (i.e. the tracking coefficient) had a *p* ≥ 0.05. Tracking analyses were adjusted for wear time, baseline age, gender and follow-up duration. Moderating effects of gender and age group were examined in separate models by adding interaction terms. For interaction terms at *p* < 0.05, tracking analyses were stratified. The variable “age group” consisted of two categories: (1) children (age at baseline and follow-up ≤12 years) and (2) transition from childhood to adolescence (baseline ≤12 years, follow-up > 12 years). Adolescence (age at baseline and follow-up > 12 years) was not included as a category because only 6 participants belonged to this group. Tracking analyses were conducted using MLwiN version 2.22.

## Results

Data from 14,098 participants was available at baseline, of which 11,284 participants (80%) had valid baseline accelerometer data. Five thousand nine hundred ninety-one participants (46% boys) provided valid accelerometer data at both baseline and at least one follow-up and were included in the analyses. Table 1 presents the participant characteristics per study. Participants were on average 10.3 years old at baseline (range: 4.7–15.0 years). Participant’s follow-up duration ranged between 0.7–8.2 years and was 2.7 years on average. Age of children at follow-up ranged from 7.6 to 17.7 years. At baseline, total sedentary time at study level ranged from 246 to 387 min/day on average. Prolonged sedentary time at study level ranged from 81 to 183 min/day on average, representing 33–50% of total sedentary time. Baseline levels of day-to-day variation in total and prolonged sedentary time at study level ranged from 41 to 55 min/day and from 32 to 48 min/day, respectively. For example, a day-to-day variation in total sedentary time of 41 min can be interpreted as, children’s daily time spent sedentary varied by 41 min from their mean weekly sedentary time.

Results of the multilevel analyses indicated that total sedentary time increased on average by 21.4 min/day [95% confidence interval (95 CI): 19.9–23.0] for each additional year of follow-up and prolonged sedentary time by 20.9 min/day [95 CI: 19.2–22.7]. Day-to-day variation in total sedentary time increased on average by 2.3 min/day [95 CI: 1.8–2.9] for each additional year of follow-up and day-to-day variation in prolonged sedentary time increased by 3.4 min/day [95 CI: 2.8–4.0].

Table 2 shows the results of the tracking analyses. Total (B = 0.48 [95 CI: 0,45; 0.50]) and prolonged (B = 0.43 [0.41; 0.45]) sedentary time tracked moderately in the total sample, while tracking of day-to-day variation in total (0.04 [0.02; 0.07]) and prolonged (0.07 [0.04; 0.09]) sedentary time was low. Regarding differences by gender, interaction terms revealed differences with a *P* < 0.05 for total and prolonged sedentary time, but these differences were small in size with slightly higher trackingcoefficients for boys (total: 0.51 [0.47; 0.55], prolonged: 0.46 [0.42; 0.49]) than for girls (total: 0.45 [0.42; 0.48], prolonged 0.42 [0.39; 0.44]. Tracking of day-to-day variation in total and prolonged sedentary time did not differ for boys and girls.

**Table 2:** Tracking of total and prolonged sedentary time and their day-to-day variation

	Total sedentary time	Prolonged sedentary time	Day-to-day variation in total sedentary time	Day-to-day variation in prolonged sedentary time
Total sample^a^ (*n* = 5991)	0.48 [0.45; 0.50] *p* < 0.0001	0.43 [0.41; 0.45] *p* < 0.0001	0.04 [0.02; 0.07] *p* < 0.0017	0.07 [0.04; 0.09] *p* < 0.0001
*Moderation by gender*^*b*^				
Boys (*n* = 2757)	0.51 [0.47; 0.55] *p* < 0.0001	0.46 [0.42; 0.49] *p* < 0.0001		
Girls (*n* = 3234)	0.45 [0.42; 0.48] *p* < 0.0001	0.42 [0.39; 0.44] *p* < 0.0001		
*Moderation by age group*^*c*^				
During childhood (*n* = 1919)	0.62 [0.57; 0.67] *p* < 0.0001			0.02 [−0.03; 0.06] *P* = 0.21
During transition from childhood to adolescence (*n* = 4050)	0.44 [0.42; 0.46] *p* < 0.0001			0.09 [0.06; 0.12] *p* < 0.0001

Values are standardized regression coefficients with 95% confidence intervals. Tracking was defined as low when the standardized coefficient was < 0.3; moderate 0.3–0.6; and high > 0.6 ‘No evidence of tracking’ was assigned when the association between baseline and follow-up levels had a *P* ≥ 0.05

^a^Analyses were adjusted for gender, follow-up duration and baseline age. ^b^ Analyses were adjusted for follow-up duration and baseline age. ^c^ Analyses were adjusted for gender and follow-up duration. When interaction terms for moderating effects had a *p* < 0.05, stratified tracking analyses are reported

Regarding differences by age group, interaction terms revealed differences with a P < 0.05 for total sedentary time and day-to-day variation in prolonged sedentary time. Stratified analyses demonstrated that tracking of total sedentary time was high during childhood (0.62 [0.57; 0.67]) and moderate during the transition from childhood to adolescence (0.44 [0.42; 0.46]). We found no evidence for tracking of day-to-day variation in prolonged sedentary time during childhood (0.02 [− 0.03; 0.06]), and low tracking in prolonged sedentary time during the transition from childhood to adolescence (0.09 [0.06; 0.12]). Tracking of prolonged sedentary time and tracking of day-to-day variation in total sedentary time was similar for both age groups (*p*-values for interaction were 0.22 and 0.14, respectively).

## Discussion

Total and prolonged sedentary time tracked moderately in young people, while tracking of day-to-day variation in total and prolonged sedentary time was low. Tracking of total sedentary time was higher during childhood than during the transition from childhood to adolescence. Tracking of total and prolonged sedentary time was slightly higher for boys than for girls. Tracking of day-to-day variation was similar for boys and girls.

Our finding that total sedentary time tracked moderately in young people is consistent with previous smaller scale studies that assessed sedentary time objectively [4, 14, 15]. In contrast, Kelly et al. [16] reported low tracking, but this study was conducted in preschool-aged children. Our finding of moderate tracking of total sedentary time is also in line with the conclusions from systematic reviews including mainly studies on TV viewing and screen time [9, 11, 12]. We found that total sedentary time increased on average by 21.4 min/day for each additional year of follow-up. A novel finding is that this increase was almost entirely due to an increase in prolonged sedentary time (> 10 min bouts; 20.9 min/day for each additional year). The study of Janssen et al. [4] reported a comparable, although slightly higher, increase in total sedentary time of 24 min per year. Tanaka et al. [5] reported in their review a higher increase of 30 min per year. Factors that may have influenced these differences in annual increase of total sedentary time are differences across studies in age of the included children, follow-up duration, included countries as well as accelerometer data reduction criteria.

We observed slightly lower tracking of sedentary time during the transition from childhood to adolescence than during childhood. This may be partly explained by children moving from primary to secondary school during the follow-up period. School transition is accompanied by changes in young people’s social and school environment that may explain the larger changes and variability in sedentary time [42, 43], thereby lowering tracking of sedentary time during the transition from childhood to adolescence. In some countries, however, such as the UK, children already attend secondary school at 12, whereas in other countries, children at this age are still in primary school. Some participants included in the ‘child’ age group would have therefore moved from primary to secondary school during follow-up, which may have attenuated the tracking estimate for the child age group.

Average total sedentary time at study level ranged from 243 to 375 min/day at baseline for boys and from 250 to 393 min/day at baseline for girls. Prolonged sedentary time at baseline ranged between 81 and 171 and 82–206 min/day, repespectively for boys and girls. Although average total and prolonged sedentary time at baseline was slightly higher for girls than for boys, tracking of total and prolonged sedentary time was similar for boys and girls, with slightly higher tracking coefficients for boys. This is in line with the reviews of Biddle et al. [9] and Pearson et al. [12] who concluded little evidence for gender differences in tracking of sedentary behaviour. One previous original study examined gender differences in tracking of accelerometer-assessed sedentary time and found similar tracking coefficients for boys and girls as well [15]. This suggest that tracking of sedentary time is similar for boys and girls.

Tracking of the day-to-day variation in total and prolonged sedentary time was low, with tracking coefficients close to zero. Thus, average values of total and prolonged sedentary time calculated over multiple days track over time, but the day-to-day patterns from which these daily averages are estimated track poorly in young people. This suggests that young people accumulate their sedentary time differently during the week but retain their rank for average total and prolonged sedentary time within the population. The poor tracking of day-to-day patterns in children implicates that sedentary behavior is variable and may therefore be targeted by interventions focusing on various types and settings of sedentary behavior throughout the day and week. Studies on the occurrence and day-to-day variability of specific types of children’s sedentary behavior are recommended to provide more insight in their sedentary patterns.

Our finding that total and prolonged sedentary time increase with age and track moderately over time suggests that sedentary behaviour already becomes habitualized during childhood. Studies with a long follow-up period are recommended to examine the degree to which young people’s total and prolonged sedentary time track into adulthood.

One key strength of this study is the large harmonised international dataset including accelerometer data. This allowed us to apply consistent data reduction decisions across the different studies included. This study is the first to examine tracking of prolonged sedentary time and the day-to-day variation in total and prolonged sedentary time. However, a limitation may be the limited representativeness of our study population, caused by multiple factors. First, the ICAD-data is not globally representative and data from the UK was relatively overrepresented in this analysis. Moreover, data from individual studies may not be nationally representative due to sampling strategies, non-response, loss to follow-up and baseline samples with valid accelerometer data being different from total study samples. In the present analysis, less than 50% of the participants with valid accelerometer data at baseline also had valid accelerometer data on at least one follow op measure. Therefore, results should be interpreted and generalised with caution.

## Conclusion

In this international sample of children and adolescents, sedentary time and prolonged sedentary time track moderately in young people while tracking of day-to-day variation in total and prolonged sedentary time was low. This suggests that young people keep their rank within the population moderately stable but the day-to-day pattern in which they accumulate their sedentary time varies over time. Total sedentary time increased on average by 21 min/day per year and this increase consisted almost completely of an increase in prolonged sedentary time.

## Data Availability

The data that support the findings of this study are available from MRC Epidemiology Unit, Cambridge. But restrictions apply to the availability of these data, which were used under license for the current study, and so are not publicly available. Data are however available from the authors upon reasonable request and with permission of MRC Epidemiology Unit, Cambridge.

## Abbreviations

95 CI: 95% confidence interval
ALSPAC: Avon Longitudinal Study of Parents And Children
BMI: Body Mass Index
CPM: counts per minute
CLAN: Children Living in Active Neighbourhoods
EYHS: European Youth Heart Study
HEAPS: Healthy Eating And Play Study
IBDS: Iowa Bone Development Study
ICAD: International Children’s Accelerometry Database
IPD meta-analyses: Individual patient data meta-analyses
MVPA: Moderate-to-vigorous intensity physical activity
PEACH: Personal and Environmental Associations with Children’s Health
SD: Standard Deviation
SPEEDY: Sport, Physical activity and Eating behaviour: Environmental Determinants in Young people
